# High expression of SLCO2B1 is associated with prostate cancer recurrence after radical prostatectomy

**DOI:** 10.18632/oncotarget.24453

**Published:** 2018-02-08

**Authors:** Tomoaki Terakawa, Eriko Katsuta, Li Yan, Nitesh Turaga, Kerry-Ann McDonald, Masato Fujisawa, Khurshid A. Guru, Kazuaki Takabe

**Affiliations:** ^1^ Department of Urology, Roswell Park Cancer Institute, Buffalo, NY, USA; ^2^ Department of Urology, Kobe University Graduate School of Medicine, Kobe, Japan; ^3^ Department of Surgical Oncology, Roswell Park Cancer Institute, Buffalo, NY, USA; ^4^ Department of Biostatistics and Bioinformatics, Roswell Park Cancer Institute, NY, USA; ^5^ Department of Surgery, University at Buffalo, Jacobs School of Medicine and Biomedical Sciences, The State University of New York Buffalo, NY, USA; ^6^ Department of Breast Surgery and Oncology, Tokyo Medical University, Tokyo, Japan; ^7^ Department of Surgery, Yokohama City University, Yokohama, Japan; ^8^ Department of Surgery, Niigata University Graduate School of Medical and Dental Sciences, Niigata, Japan

**Keywords:** prostate cancer, SLCO2B1, recurrence, EMT, OATP

## Abstract

Solute carrier organic anion (SLCO) gene families encode organic anion transport proteins, which are transporters that up-take a number of substrates including androgens. Among them, high expression of SLCO2B1 is known to associate with the resistance to androgen deprivation therapy in prostate cancer (PCa). We hypothesized that high expression of SLCO genes enhances PCa progression by promoting the influx of androgen. Here, we demonstrated the impact of the expression levels of SLCO2B1 on prognosis in localized PCa after radical prostatectomy (RP) utilizing 494 PCa cases in The Cancer Genome Atlas (TCGA). SLCO2B1 high expression group showed significantly worse Disease-free survival (DFS) after RP (*p* = 0.001). The expression level of SLCO2B1 was significantly higher in advanced characteristics including Gleason Score (GS ≤ 6 vs GS = 7; *p* = 0.047, GS = 7 vs GS ≥ 8; *p* = 0.002), pathological primary tumor (pT2 vs pT3/4; *p* < 0.001), and surgical margin status (positive vs negative; *p* = 0.013), respectively. There was a significant difference in DFS between these two groups only in GS ≥ 8 patients (*p* = 0.006). Multivariate analysis demonstrated that only SLCO2B1 expression level was an independent predictor for DFS after RP in GS ≥ 8. SLCO2B1 high expressed tumors in GS ≥ 8 not only enriched epithelial mesenchymal transition (EMT) related gene set, (*p* = 0.027), as well as Hedgehog (p < 0.001), IL-6/JAK/STAT3 (*p* < 0.001), and K-ras signaling gene sets (*p* < 0.001), which are known to promote EMT, but also showed higher expression of EMT related genes, including N-cadherin (p = 0.024), SNAIL (*p* = 0.001), SLUG (*p* = 0.001), ZEB-1 (*p* < 0.001) and Vimentin (*p* < 0.001). In conclusion, PCa with high expression of SLCO2B1 demonstrated worse DFS, which might be due to accelerated EMT.

## INTRODUCTION

Prostate cancer (PCa) is the most common malignant disease and the second leading cause of cancer related death among men in western industrialized countries [1]. Radical prostatectomy (RP) is the gold standard treatment for localized PCa, however, approximately 30% of the cases develop recurrence after RP despite advances in surgical techniques [2]. Several pathological features such as positive surgical margins, extracapsular invasion, or seminal vesicle invasion were found to be associated with recurrence after RP [3, 4]. Pathological Gleason score (GS) has also been recognized as one of the most reliable prognostic factors predicting recurrence after RP [4–7].

To reduce the risk of recurrence, adjuvant treatment including radiotherapy or hormonal therapy has been suggested for patients with high risk pathological features [3, 8, 9]. However, the optimal adjuvant therapy for these high-risk patients is still a subject of continuous debate. Indeed, only 10–35% of the patients with extracapsular invasion develop recurrence [10–12] and the recurrence rate after RP differs immensely between studies [13]. These data suggest that current adjuvant therapies may include considerable overtreatment and indicate that a prognostic biomarker to precisely identify patients at high-risk of recurrence after RP is in urgent need.

Solute carrier organic anion (SLCO) gene families encode organic anion transport proteins (OATP), which are membrane transporters widely expressed in the human body that influx numerous compounds and drugs including androgens [14–16]. Several investigations have shown that SLCO genes, SLCO2B1 and SLCO1B3, mediate the uptake of androgen into PCa cells. SLCO2B1 transports one of the adrenal androgens, dehydroepiandrosterone sulfate (DHEAS), which is a precursor to the most potent androgen receptor into normal prostate and PCa cell. SLCO2B1 expression levels have been shown to correlate with DHEAS uptake in PCa cell lines [17]. On the other hand, SLCO1B3 transports testosterone, which is essential for progression of PCa [14, 18]. In agreement, the expression of these genes has been shown to be associated with the resistance to androgen deprivation therapy, which is the standard treatment for PCa [19, 20]. SLCO2B1 has been shown to be significantly increased in metastatic tissue, compared to primary tissue in PCa [21], and polymorphisms of SLCO2B1 gene is associated with shorter time to progression and overall survival (OS) in patients with metastatic PCa receiving androgen deprivation therapy [17, 22]. These findings suggest that SLCO2B1 expression is associated with the progression of PCa.

However, the significance of SLCO2B1 gene expression on the recurrence after RP in PCa has not been investigated. We hypothesized that the expression level of SLCO genes will affect the progression of PCa and lead micro-metastasis by promoting the influx of androgen. In the present study, we investigated the impact of the expression level of SLCO2B1 on patient survival in localized PCa who underwent RP.

## RESULTS

### The cancer genome atlas (TCGA) PCa patient cohort

**Figure 1:**
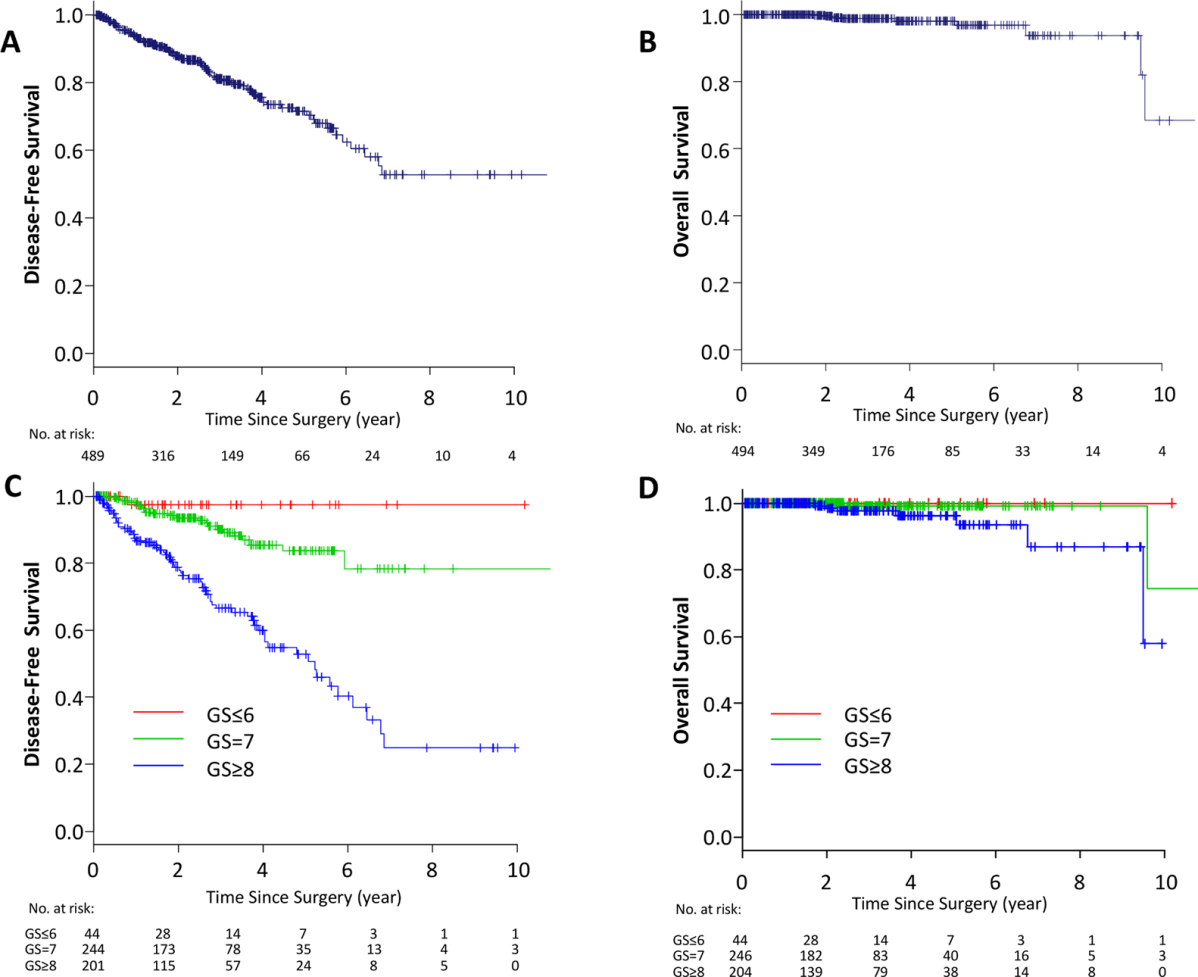
Kaplan–Meier estimate of survival in TCGA PCa patients DFS (**A**) and OS (**B**) in whole TCGA PCa patients. DFS (**C**) and OS (**D**) classified by GS in whole TCGA PCa patients. Red line; GS ≤ 6, green line; GS = 7, blue line GS ≥ 8.

In whole TCGA PCa (PRAD) cohort, there were 494 PCa cases with gene expression and survival data to analyze Disease-free survival (DFS) (*n* = 489) and OS (*n* = 494). The mean age of the cohort was 61.0 + 6.8 years old. The patient proportion of each pathologic primary tumor status for pT2, pT3a, pT3b and pT4 was 185 (37.4%), 158 (32%), 135 (27.3%) and 10 (20.2%), respectively (Table 1). DFS rates of 3, 5 and 10-year were 81.0%, 71.5% and 52.8%, respectively (Figure 1A). OS rates of 3, 5 and 10-year were found to be 98.7%, 98.0% and 68.3%, respectively (Figure 1B). According to GS of the prostatectomy specimens, 8.9% of patients had GS ≤ 6, followed by 49.8% and 41.3% of patients with GS = 7 and GS ≥ 8, respectively (Table 1). Five-year DFS rates were 97.4%, 78.4% and 25.0% in GS ≤ 6, GS = 7 and GS ≥ 8, respectively (Figure 1C). OS rates at 10-years were found to be 100%, 74.4% and 57.9% in the patients with GS ≤ 6, GS = 7 and GS ≥ 8, respectively (Figure 1D). These data demonstrate that patients in TCGA have more advanced disease compared to the recent reports from high volume institutions or multi-institutional studies since the proportion of pT2 patients is less than 40% in this cohort, whereas it surpassed 60% in recent reports [23–26]. The fact that patients with more advanced disease are included in this series leads the results of the higher proportion of high GS and inferior DFS comparing to recent reports.

**Table 1:** Demographics and PCa characteristics

	Patients (*n* = 494)
Age (y.o.)^†^	61.0 ± 6.8
GS (%)	
≤6	44 (8.9%)
7	246 (49.8%)
≥8	204 (41.3%)
Pathological Primary tumor: pT (%)	
pT2	185 (37.4%)
pT3a	158 (32.0%)
pT3b	135 (27.3%)
pT4	10 (20.2%)
Regional Lymph Node: *N* (%)	
N0	343 (69.4%)
N1	79 (16.0%)
Surgical margin status (%)	
negative	313 (63.4%)
positive	152 (30.8%)
Adjuvant radiation therapy (%)	
no	246 (49.8%)
yes	40 (8.1%)

^†^: mean ± SD

### High expression of SLCO2B1, but not SLCO1B3, is associated with worse DFS after RP in PCa

**Figure 2:**
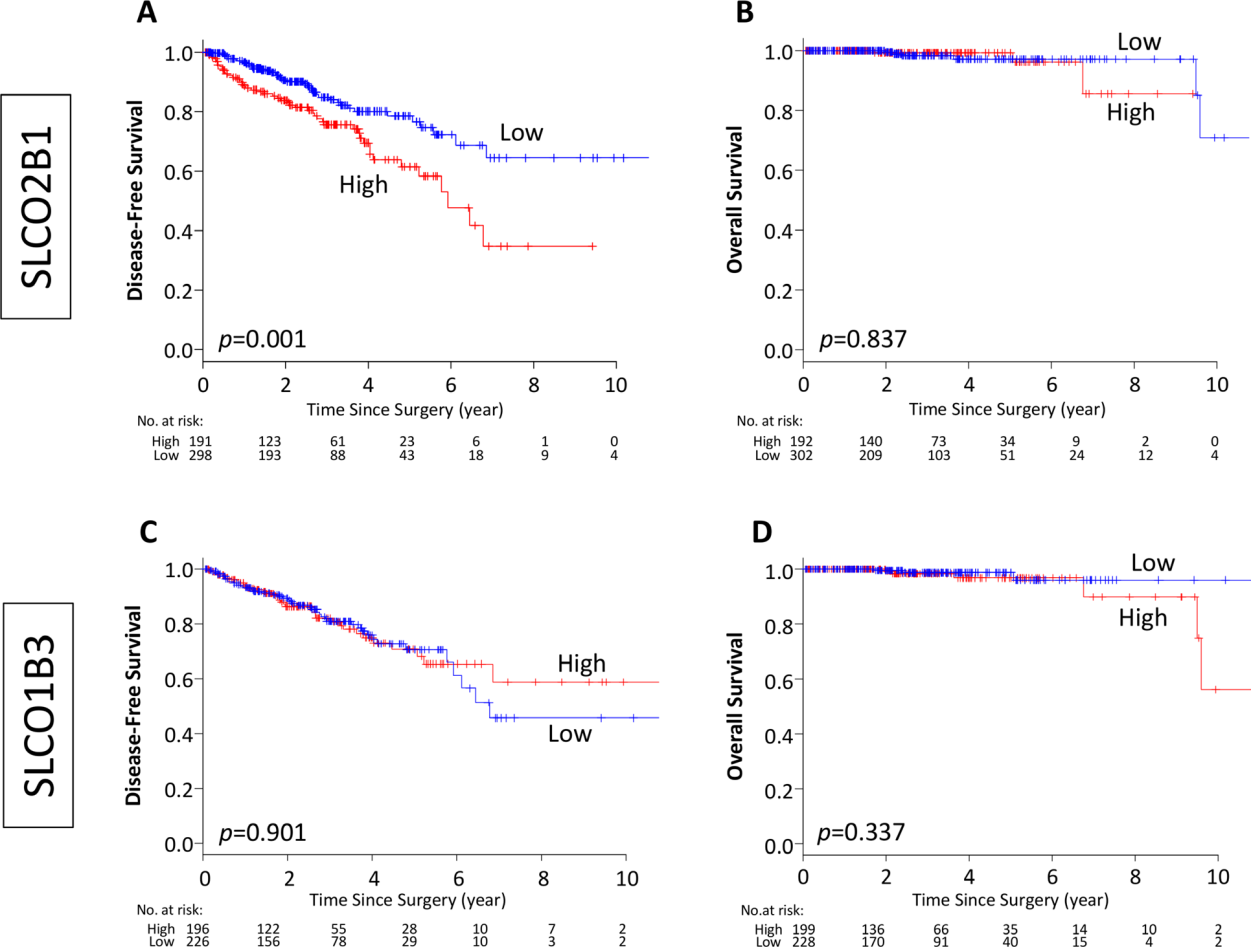
Kaplan–Meier estimate of disease-free and overall survival by dichotomized of SLCO2B1 and SLCO1B3 expression in TCGA PCa patients (**A**, **B**) Classified by SLCO2B1 expression level, (**C**, **D**) classified by SLCO1B3 expression level. Red line; high expression, blue line; low expression of each genes.

First, we investigated the association of SLCO genes, SLCO2B1 and SLCO1B3, expression and prognosis of the PCa patients who underwent RP utilizing TCGA cohort. There are two patients (0.4%) who have SLCO2B1 mutation and no patient has SLCO1B3 mutation, which suggests that there is minimal effect of mutation status on RNA expression. Patients were dichotomized into two groups, and the classification cutoff was determined by the mean value of the gene expression levels. Out of 494 cases, 192 and 302 patients were classified as SLCO2B1 high and low expression groups, respectively. We found that high expression of SLCO2B1 was associated with worse DFS after RP (*p* = 0.001), whereas there was no significant difference in OS between these two groups (*p* = 0.837) (Figure 2A, 2B). SLCO1B3 is another member of the SLCO gene family that influxes androgens into PCa cells. In contrast to SLCO2B1, there was no significant difference in OS or DFS between high and low expression levels of SLCO1B3 (Figure 2C, 2D). The findings that high SLCO2B1 expressing tumors have worse DFS but no difference in OS were validated using the median cutoff in TCGA dataset (Supplementary Figure 1A, 1B).

### High SLCO2B1 expression is associated with aggressive pathological features

**Figure 3:**
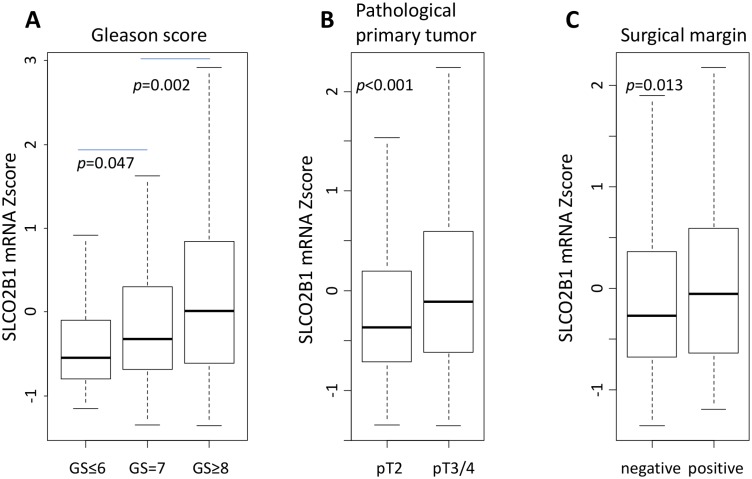
The expression level of SLCO2B1 according to pathological factors in TCGA PCa patients (**A**) GS. (**B**) pathological primary tumor status (pT). (**C**) surgical margin status.

SLCO2B1 expression levels were analyzed in different GS, pT status and surgical margin status. GS is one of the most reliable pathological parameters to determine aggressiveness of PCa, and positive surgical margins suggest the aggressive biology of a tumor. Interestingly, SLCO2B1 expression levels were all equivocally elevated in more advanced histological features such as GS (GS ≤ 6 vs GS = 7; *p* = 0.047, GS = 7 vs GS ≥ 8; *p* = 0.002), pT (pT2 vs pT3/4; *p* < 0.001), and surgical margin status (negative vs positive; *p* = 0.013) (Figure 3).

### High expression of SLCO2B1 is a prognostic biomarker in GS high, but not in GS low tumors

**Figure 4:**
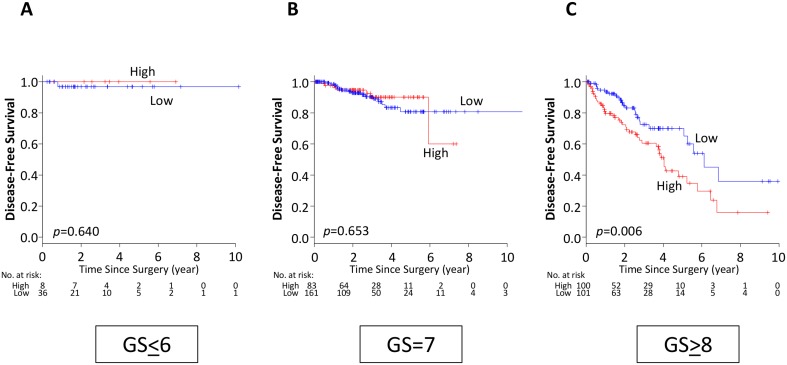
DFS classified by SLCO2B1 expression level in the subgroups according to GS in TCGA PCa patients (**A**) GS ≤ 6, (**B**) GS = 7, (**C**) GS ≥ 8. Red line; high expression, blue line; low expression of SLCO2B1.

Since SLCO2B1 expression was significantly higher in GS high tumors, we hypothesized that SLCO2B1 expression was associated with tumor aggressiveness in GS high tumors. Strikingly, high expression of SLCO2B1 was significantly associated with worse DFS only in GS ≥ 8 population (*p* = 0.006), and not in the other GS (GS ≤ 6; *p* = 0.640, GS = 7; *p* = 0.653) (Figure 4). These findings indicate that SLCO2B1 expression could be a prognostic biomarker of recurrence after RP in GS ≥ 8 patients. The findings that high SLCO2B1 expressing tumors have worse DFS in GS ≥ 8 but not in GS ≤ 7 patients were validated using the median cutoff in TCGA dataset (Supplementary Figure 1C–1E).

### Clinicopathological features of patients with SLCO2B1 high expression among GS ≥ 8 tumors

Since high expression of SLCO2B1 associated with cancer recurrence only in GS ≥ 8 patients, clinicopathological features were compared between SLCO2B1 high and low expression groups in PCa patients with GS ≥ 8 to investigate whether certain populations associated with SLCO2B1 expression (Table 2). Interestingly, there was no significant difference in age, pT status (pT2 vs pT3/4), lymph node metastasis, surgical margin status, and postoperative radiation therapy between these two groups.

**Table 2:** Clinicopathological characteristics of SLCO2B1 high and low in the PCa patients with GS ≥ 8

	High (*n* = 101)	Low (*n* = 103)	*p*
Age (y.o.)^†^	62.2 ± 5.6	62.6 ± 7.3	0.666
Pathological Primary tumor: pT			0.309
pT2	11	17	
pT3a	25	37	
pT3b	60	45	
pT4	5	2	
Regional Lymph Node: N			0.648
N0	64	63	
N1	30	35	
Surgical Margin			0.564
negative	45	48	
positive	53	46	
Adjuvant RT			0.570
no	50	61	
yes	19	18	

^†^: mean ± SD.

### SLCO2B1 is the only independent predictor for DFS in the patients with GS ≥ 8

We investigated the influence of SLCO2B1 expression in DFS compared to other clinical or pathological factors known to impact PCa recurrence by univariate and multivariate analyses using Cox proportional hazards regression. Pathological primary tumor status (*p* = 0.048), surgical margin status (*p* = 0.030) and SLCO2B1 expression (*p* = 0.007) were significant prognostic factors in the univariate analysis. Interestingly, multivariate analysis revealed that high expression of SCLO2B1 was the only independent prognostic factor for DFS (*p* = 0.022) (Table 3).

**Table 3:** Univariate and multivariate analyses for independent DFS predictor of PCa patients with GS ≥ 8

	Univariate analysis			Multivariate analysis		
Clinicopathological factor	HR	(95% CI)	*p*	HR	(95% CI)	*p*
Age (>70 vs <70)	0.884	(0.352−2.217)	0.792			
pT (pT3,4 vs pT2)	2.518	(1.007−6.299)	0.048^*^	2.223	(0.867−5.679)	0.096
N (N1 vs N0)	0.852	(0.495−1.468)	0.564			
Surgical margin (positive vs negative)	1.750	(1.057−2.896)	0.030^*^	1.607	(0.96−2.692)	0.071
Adjuvant Radiation (yes vs no)	1.038	(0.55−1.959)	0.909			
SLCO2B1 (High vs Low)	1.991	(1.204−3.294)	0.007^*^	1.829	(1.093−3.061)	0.022^*^

^*^:*p* < 0.05.

### Gene expression between high and low expression of SLCO2B1 in PCa with GS ≥ 8

To investigate the mechanism of how high expression of SLCO2B1 is associated with high recurrence rate in the patients with GS ≥ 8 after RP, Gene Set Enrichment Analysis (GSEA) was conducted. GSEA demonstrated that numerous genes were differently expressed between high and low expression of SLCO2B1. 2908 genes were upregulated with score >3.0 of GSEA, and 1411 genes were downregulated in the SLCO2B1 high expression group with score < −3.0 of GSEA. The heatmap of the top 50 upregulated and downregulated genes are shown in Supplementary Figure 2, as well as the list of genes (Supplementary Table 1).

### Key cancer signaling pathways related gene sets were enriched in SLCO2B1 high with GS ≥ 8

**Figure 5:**
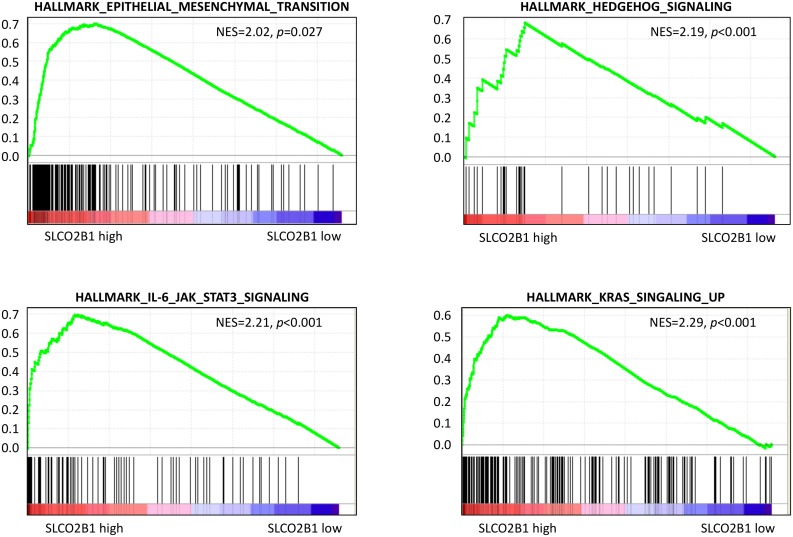
GSEA between SLCO2B1 high and low expression in patients with GS ≥ 8

GSEA demonstrated that among 50 hallmark gene sets, 11 gene sets were significantly enriched in SLCO2B1 high expressed tumors with family wise error rate (FWER) *p* < 0.05 (Supplementary Table 2), and 1 gene set was significantly enriched in SLCO2B1 low with FWER *p* < 0.05 (Supplementary Table 3). Of those, SLCO2B1 high expressed tumors enriched a gene set related to epithelial mesenchymal transition (EMT), which is known to be one of the major mechanisms of metastasis and recurrence (Normalized enrichment score; NES = 2.02, FWER *p* = 0.027), as well as some of the pathway related gene sets known to promote EMT [27–30], including Hedgehog signaling gene set (NES = 2.19, FWER *p* < 0.001), IL-6/JAK/STAT3 signaling gene set (NES = 2.21, FWER *p* < 0.001) and K-ras signaling gene set (NES = 2.29, FWER *p* < 0.001) (Figure 5). These gene sets were also enriched in SLCO2B1 high expressed tumors in GS ≤ 7 tumors (Supplementary Figure 3).

### High expression of SLCO2B1 significantly upregulated EMT related gene expression in PCa with GS ≥ 8

**Figure 6:**
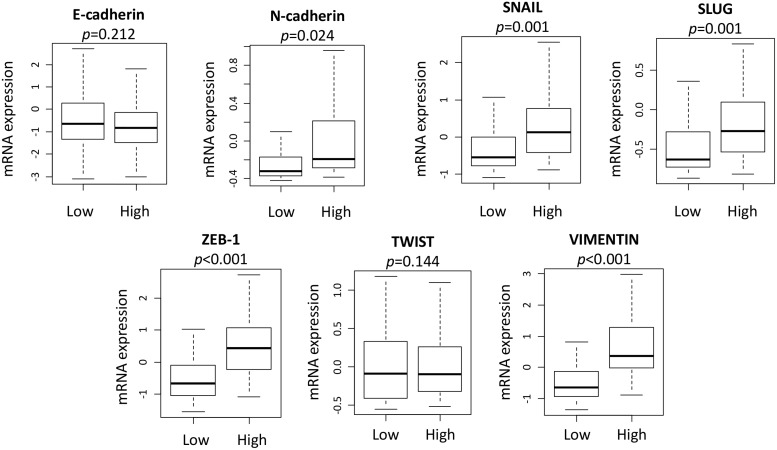
EMT related genes expression comparison between SLCO2B1 high and low in patients with GS ≥ 8

In agreement with the GSEA results, the expression level of some EMT related genes, including N-cadherin (*p* = 0.024), SNAIL (*p* = 0.001) and SLUG (*p* = 0.001), ZEB-1 (*p* < 0.001) and Vimentin (*p* < 0.001), were significantly higher in SLCO2B1 high group in GS ≥ 8 (Figure 6). This result is in sync with our previous publication that EMT is strongly associated with recurrence after RP [31, 32]. Therefore, one of the mechanisms responsible for the worse prognosis of SLCO2B1 high expression after RP may be due to promoted EMT.

## DISCUSSION

In this study, we found that high expression of SLCO2B1, which mediates the uptake of DHEAS into PCa cells, is associated with advanced pathological features in PCa including GS as well as worse DFS after RP particularly among high GS patients. Furthermore, we elucidated that high SLCO2B1 expression tumors enriched EMT related gene sets as well as key cancer signaling pathways related gene sets that promote EMT. These results imply that this may be one of the mechanisms responsible for the worse prognosis after RP in SLCO2B1 high expressed tumor. To our knowledge, this is the first report showing the clinical relevance of SLCO2B1 expression on the recurrence after RP.

Among a number of SLCO family genes, SLCO2B1 and SLCO1B3 have been implicated in PCa progression, as they both influx androgens such as testosterone and DHEAS which are important precursors to the androgen receptor. A number of studies have shown that SLCO2B1, which encodes OATP2B1, influx endogenous steroids such as DHEAS in PCa. DHEAS uptake has been shown to be dependent on the expression level of SLCO2B1. Greater expression of SLCO2B1 leads to increased DHEAS transport into cells in PCa cell lines [17], and overexpression of SLCO2B1 increases tumor DHEAS accumulation in a PCa murine model [33]. These findings suggest that SLCO2B1 has an important role in transportation of DHEAS in PCa. Furthermore, genetic variation of SLCO2B1 is associated with resistance to androgen deprivation therapy as well as OS of PCa, whereas there was no difference in recurrence after RP [17, 21, 22]. Among the various members of SLCO gene families, SLCO1B3 which also transports androgen is associated with resistance to hormonal treatment in PCa [21, 22]. However, in our results, SLCO1B3 expression was not associated with recurrence after RP, while SLCO2B1 was significantly related and has been shown to be the most important predictor of recurrence after surgery in patients with high GS, compared to other known factors.

To investigate underlying mechanisms, we found that high expression of SLCO2B1 is associated with EMT. It has been shown that the expression of EMT markers is significantly associated with outcomes following RP in localized PCa [31, 32]. We previously reported that the expression levels of EMT markers in RP specimens, Twist and Vimentin in particular, was significantly related to recurrence in addition to conventional prognostic parameters [31]. It has also been reported that high expression of Vimentin is a prognostic marker of shorter recurrence-free survival in PCa [32]. In this study, GSEA demonstrated that SLCO2B1 high expression group also enriched gene sets that promote EMT, including the sonic hedgehog pathway, IL-6/JAK/STAT3 pathway and K-Ras signaling pathway related gene sets. Sonic hedgehog pathway has been shown to drive EMT via upregulating N-cadherin and Vimentin in PCa [27]. IL-6/JAK/STAT3 pathway was shown to activate EMT through upregulation of Twist, N-cadherin and Vimentin in some types of cancer [28, 29]. Moreover, EMT has been reported to be promoted by the K-ras signaling pathway [30]. Considering these previous reports and our GSEA results, high expression of SLCO2B1 appears to activate EMT through the upregulation of sonic hedgehog, IL-6/JAK/STAT3 and K-ras signaling pathways. In agreement, DHEA, one of the major metabolites from DHEAS, has been shown to accelerate EMT through E-cadherin suppression and the induction of N-cadherin and Vimentin in PCa [34]. Taken together, it is possible that high expression of SLCO2B1 accelerates the influx of DHEAS, which is then metabolized to the more active DHEA. Accumulated DHEA induces EMT by the activated sonic hedgehog pathway, IL-6/JAK/STAT3 pathway and K-ras signaling pathway. EMT may promote dissemination and subclinical metastasis prior to RP, leading to recurrence after RP. Although EMT related gene sets were also enriched in SLCO2B1 high group in GS ≤ 7 patients, these patients were not associated with worse DFS. Considering the result that the expression levels of SLCO2B1 is much higher in GS ≥ 8 tumors compared to in GS ≤ 7 tumors, we speculate that higher expression of SLCO2B1 is required to accelerate this mechanism leading to recurrence in the patients after RP.

Statins, a class of cholesterol-lowering medications that inhibit 3-hydroxy-3-methl-glutaryl-coenzyme A reductase, are also substrates of SLCO2B1 and have been shown to act as an anticancer drug in various types of cancers [35]. In PCa, clinical studies have shown the controversial results in statin treatment after RP [36–39]. It has been reported that DHEAS uptake is inhibited by statins in PCa cell lines [40], and statin treatment decreased castration resistant progression with lower intra-tumoral androgen levels in murine models [41]. These results suggest that the biological mechanism of statins in PCa may be competitive inhibition of the uptake of DHEAS with SLCO2B1 encoded transporters, OATP2B1. Together with our results, we believe that a randomized clinical trial using high SLCO2B1 expression as a predictive biomarker for statin treatment for GS high PCa to prevent recurrence after RP is warranted.

Although the present study demonstrates novelty, it has limitations. First, this study was conducted using only one publically available dataset. It will be ideal to compare SLCO2B1 expression level between primary tumor with and without metastasis because EMT is a well-known mechanism in metastatic disease and cancer progression, and validate SLCO2B1 expression level and recurrence using another cohort. However, we were unable to find a dataset which has SLCO2B1 expression level of primary tumor with and without metastasis, as well as SLCO2B1 expression level with patient recurrence-free survival. Secondly, this study is based only on the gene expression of the primary tumor in TCGA cohort and not based on any *in vitro* and *in vivo* experiments. In order to determine the role of SLCO gene or to elucidate its molecular mechanism, the experimental approach is needed.

In conclusion, PCa with high expression of SLCO2B1 in high GS tumors demonstrated worse DFS, which might be due to accelerated EMT. Further studies are needed to determine the clinical application of SLCO2B1 levels.

## MATERIALS AND METHODS

### Data acquisition and pre-processing

There are 498 patients who underwent RP for non-metastatic disease in the PCa cohort of TCGA. Out of 498, 2 patients with neoadjuvant hormone therapy were excluded from our analysis. Gene expression data and survival data were available in total 494 patients. The gene expression level quantification data (mRNA expression z-score of RNA-seq) were downloaded through cBioPortal and used as previously described [42–44]. Patients were classified into SLCO2B1 high and low groups according to their gene expression level, the cutoff was determined as mean value (0.025). DFS was calculated from time of diagnosis to either biochemical recurrence or radiological tumor recurrence/metastasis.

### GSEA of TCGA cohort

GSEA was performed on TCGA cohort using software provided by the Broad Institute (http://software.broadinstitute.org/gsea/index.jsp), as described before [45–47]. We classified the patients into two groups according to SLCO2B1 expression using same cutoff value (mean: 0.025).

### Statistical analysis

The prognosis differences between SLCO2B1 high and low groups, including OS and DFS, were analyzed using Kaplan-Meier method with log-rank test. Statistical comparisons of the clinicopathological characteristics for significance were performed by the chi-square test or the Fisher exact test, and a Student *t*-test was used to analyze the differences between continuous values. In multivariate analysis, Cox proportional hazards regression method was used in order to identify the most significant independent prognostic factors. In all analysis, a two-sided *p* < 0.05 was considered as statistically significant. All statistical analyses were performed using R software (http://www.r-project.org/) and Bioconductor (http://bioconductor.org/).

## SUPPLEMENTARY MATERIALS FIGURES AND TABLES







## Abbreviations

SLCO: Solute carrier organic anion
PCa: prostate cancer
RP: radical prostatectomy
PSA: prostate specific antigen
OATP: organic anion transport proteins
GS: Gleason score
DHEAS: dehydroepiandrosterone sulfate
TCGA: The Cancer Genome Atlas
DFS: disease-free survival
OS: overall survival
GSEA: Gene Set Enrichment Analysis
FWER: family wise error rate
EMT: epithelial-mesenchymal transition.
