# Supplementary material for: High expression of SLCO2B1 is associated with prostate cancer recurrence after radical prostatectomy
Source: Oncotarget. 2018 Feb 8;9(18):14207–18. doi: 10.18632/oncotarget.24453 (PMC5865664; doi:10.18632/oncotarget.24453)
Supplement: Supplementary file 1 [file oncotarget-09-14207-s001.pdf]

High expression of SLCO2B1 is associated with prostate cancer recurrence after radical prostatectomy

SUPPLEMENTARY MATERIALS

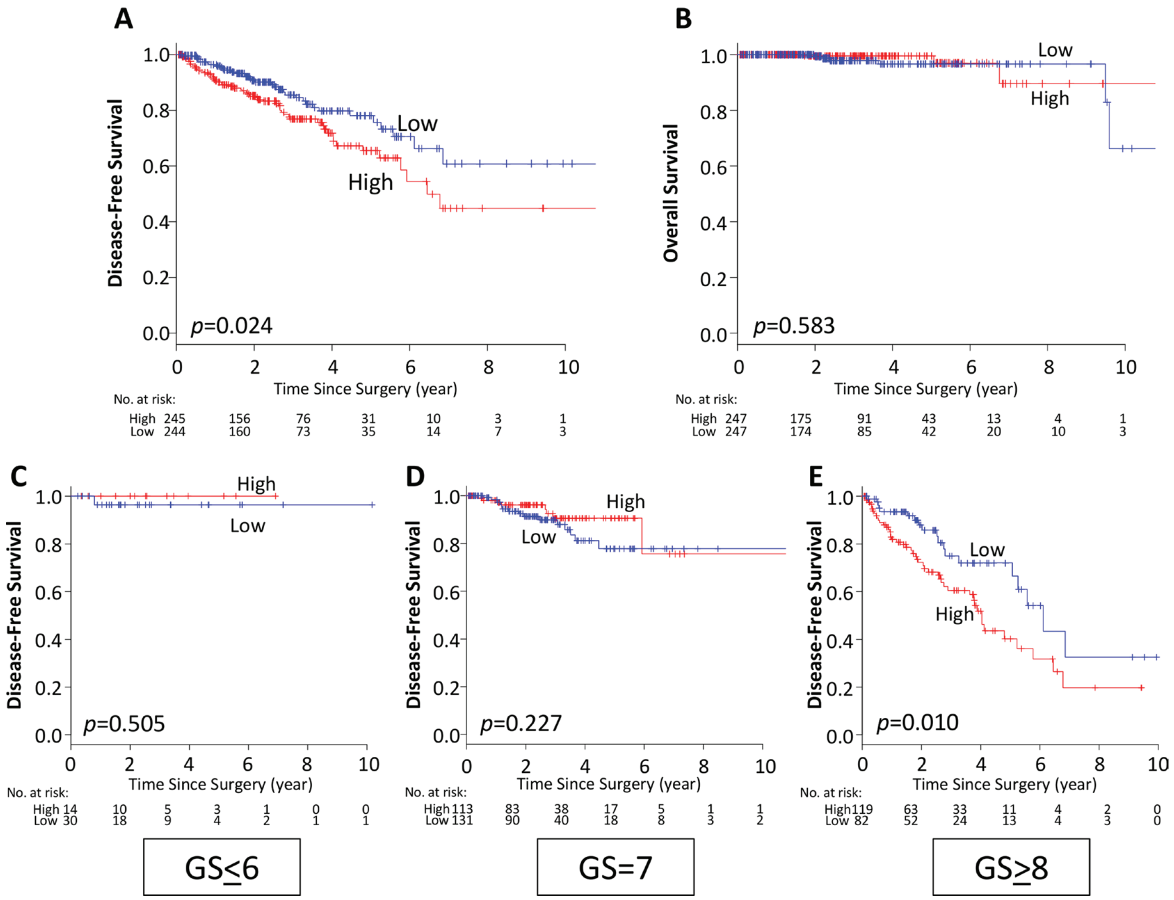

Supplementary Figure 1:SLCO2B1 expression and patient's survival validation using another cut off. (**A**) DFS and (**B**) OS classified by SLCO2B1 expression using median cut off in whole TCGA PCa patients. DFS classified by SLCO2B1 expression level using median cutoff in the subgroups according to GS in TCGA PCa patients, (**C**) GS < 6, (**D**) GS = 7, (**E**) GS > 8. Red line; high expression, blue line; low expression of SLCO2B1.

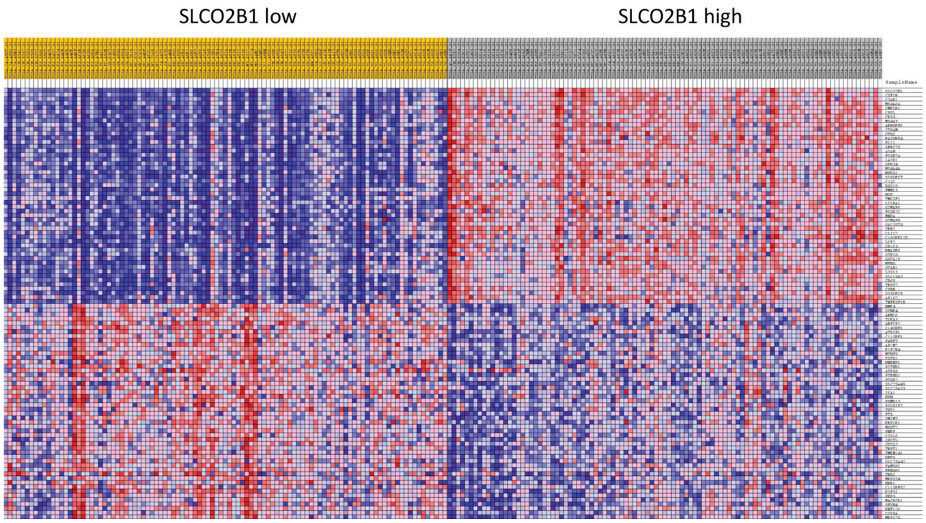

Supplementary Figure 2: Heatmap of differently expressed genes between SLCO2B1 high and low expressed tumors in TCGA PCa patients with GS ≥ 8.

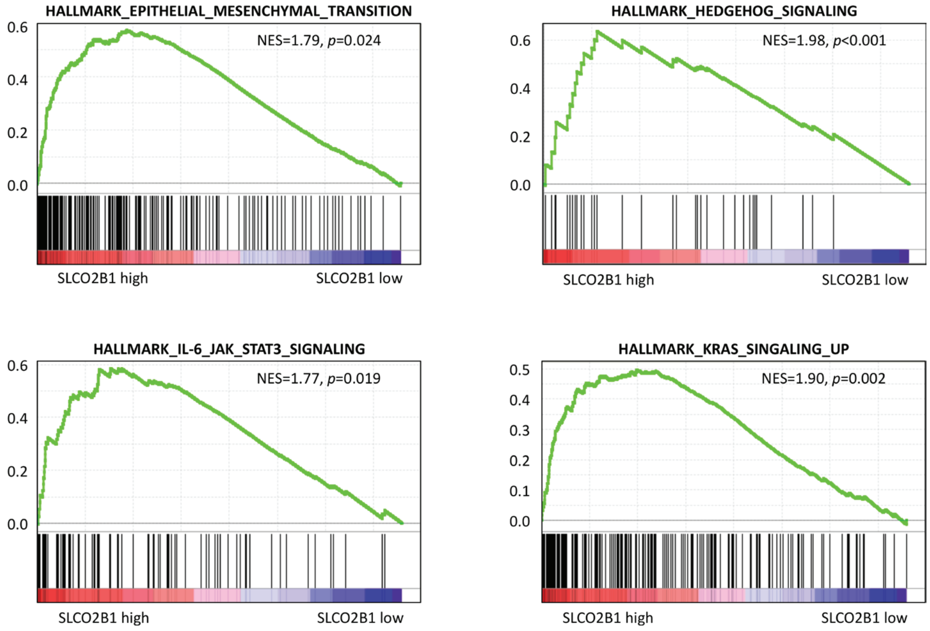

Supplementary Figure 3: GSEA between SLCO2B1 high and low expression in patients with GS < 7.

Supplementary Table 1: Top 50 upregulated and downregulated genes in SLCO2B1 high expression group. See Supplementary_Table_1

Supplementary Table 2: Gene sets enriched in SLCO2B1 high with GS > 8. See Supplementary_Table_2

Supplementary Table 3: Gene sets enriched in SLCO2B1 low tumors

Gene Set	SIZE	ES	NES	NOM *p*-val	FDR *q*-val	FWER *p*-val
HALLMARK_OXIDATIVE_PHOSPHORYLATION	197	–0.6912735	–2.1250546	<0.001	0.004639816	0.007
HALLMARK_MYC_TARGETS_V1	199	–0.6513375	–1.9108084	0.005780347	0.02820259	0.068
HALLMARK_MYC_TARGETS_V2	58	–0.6725976	–1.7576495	0.025540275	0.08073441	0.219
HALLMARK_UNFOLDED_PROTEIN_RESPONSE	109	–0.44493386	–1.6655595	0.019646365	0.108996086	0.352
HALLMARK_DNA_REPAIR	143	–0.41944596	–1.5612028	0.05668016	0.15990826	0.514
HALLMARK_FATTY_ACID_METABOLISM	157	–0.35975504	–1.4081328	0.07984032	0.2764803	0.762
HALLMARK_CHOLESTEROL_HOMEOSTASIS	73	–0.38542786	–1.340715	0.16116504	0.31775385	0.844
HALLMARK_ADIPOGENESIS	195	–0.32450396	–1.3237405	0.12269939	0.2971208	0.857
HALLMARK_PEROXISOME	102	–0.29288545	–1.2056745	0.19960861	0.40651175	0.934
HALLMARK_MTORC1_SIGNALING	198	–0.33253804	–1.1907779	0.27111983	0.3843927	0.939
HALLMARK_ANDROGEN_RESPONSE	98	–0.33752018	–1.1542249	0.29865125	0.3953616	0.96
HALLMARK_GLYCOLYSIS	198	–0.22325516	–0.9471251	0.49122807	0.65294296	0.998
HALLMARK_REACTIVE_OXIGEN_SPECIES_PATHWAY	46	–0.26951456	–0.92822915	0.55009824	0.6305486	0.999
HALLMARK_PROTEIN_SECRETION	95	–0.2084143	–0.6969045	0.8007737	0.93080914	1
HALLMARK_E2F_TARGETS	197	–0.21470378	–0.6038966	0.7598425	0.96377546	1
HALLMARK_G2M_CHECKPOINT	196	–0.1173306	–0.3544897	0.993988	0.9960832	1

ES: enrichment score, NES: normalized enrichment score, FDR: false discovery rate, FWER: family wise error rate.
